# A Comparison of Order-Disorder in Several Families of Cubic Oxides

**DOI:** 10.3389/fchem.2021.719169

**Published:** 2021-09-01

**Authors:** T. Subramani, A. Voskanyan, K. Jayanthi, M. Abramchuk, A. Navrotsky

**Affiliations:** School of Molecular Sciences and Navrotsky Eyring Center for Materials of the Universe, Arizona State University, Tempe, AZ, United States

**Keywords:** oxides, pyrochlores, fluorite, disorder, energetics

## Abstract

Order-disorder on both cation and oxygen sites is a hallmark of fluorite-derived structures, including pyrochlores. Ordering can occur on long- and short-range scales and can result in persistent metastable states. In various cubic oxide systems, different types of disorder are seen. The purpose of this paper is to review and compare the types and energetics of order-disorder phenomena in several families of cubic oxides having pyrochlore, weberite, defect fluorite, perovskite, rocksalt, and spinel structures. The goal is to better understand how structure, composition, and thermodynamic parameters (enthalpy and entropy) determine the feasibility of different competing ordering processes and structures in these diverse systems.

## Introduction

Over the past two decades, there has been intense interest in defect chemistry, disorder, solid solution formation, and phase transitions in oxide pyrochlores (Glerup et al., 2001; Shlyakhtina et al., 2004; Liu et al., 2004; Knee et al., 2005; Mandal et al., 2006; Matsuhira et al., 2007; Zhang et al., 2008; Clements et al., 2011; Wang et al., 2011; de los Reyes et al., 2013; Zhang et al., 2013; Zhang et al., 2014; Popov et al., 2016; Paul et al., 2016; Rittman et al., 2017a; Rittman et al., 2017b; Simeone et al., 2017; Ponnilavan et al., 2019; Popov et al., 2020; Marlton et al., 2021; Panghal et al., 2021). Because the pyrochlore structure is a derivative of fluorite, it belongs to a large family of related structures. The interplay of short- and long-range order, especially in radiation-damaged pyrochlores, has drawn extensive attention as has amorphization and its recovery on annealing (Wang et al., 1998; Wang et al., 1999; Meldrum et al., 2001; Lian et al., 2002; Ewing et al., 2003; Lian et al., 2003a; Lian et al., 2004; Patel et al., 2008a; Patel et al., 2008b; Lang et al., 2009; Yudinsev et al., 2009; Lang et al., 2010; Xie et al., 2015; Shamblin et al., 2016a; Shamblin et al., 2016b; Kumari et al., 2016; Yang et al., 2017; Chung et al., 2018a; Chung et al., 2018b; Shamblin et al., 2018; Chung et al., 2019; Sherrod et al., 2021). Yet in a broader sense, pyrochlores are but one family of oxide materials.

Focusing on cubic structures, other families include defect fluorite ionic conductors such as yttria-stabilized zirconia (YSZ), weberites (both oxides and fluorides), rocksalt-based cation conducting oxides, perovskites, and spinels, all of which form the basis of numerous functional materials. Each of these families shows distinct and characteristic defect chemistry and order-disorder behavior.

Each simple structure (aristotype) is the parent of more complex structures showing ordering on anion and/or cation sublattices and the formation of layered structures. Modern diffraction and spectroscopy enable characterization of order on different length scales, with short-range order producing lower symmetry locally being detected increasingly often in nominally cubic structures (Radhakrishnan et al., 2011; Shamblin et al., 2016a; Shamblin et al., 2016b; Martel et al., 2017; Shamblin et al., 2018; Moran et al., 2019; Drey et al., 2020). The details of ordering are different in each structural family. Though at equilibrium at a given pressure, temperature, and composition, defect formation and order-disorder produce, by definition, the state of lowest Gibbs free energy, this macroscopic truism begs the question of why: why, in a structural sense, is one set of defects predominant while others are not, and why, in different structure types, does one get characteristic and distinct sets of defects. Such questions can be addressed by first-principles calculations (e.g., density functional theory and molecular dynamics) or by calculations using semiempirical interatomic potential. The results, though identifying the lowest energy configurations, are hard to compare for different structures and do not immediately give physical insight, in terms of identifiable structural parameters, to the question of why (Gunn et al., 2012; Xiao et al., 2015; Perriot et al., 2016; Solomon et al., 2016; Pilania et al., 2019; O’Quinn et al., 2020). Furthermore, many of the complex structures have too many elements and too large unit cells for meaningful computations. Computational approaches to different structures (possible or nonexistent) polymorphs for a given composition have also been very limited.

For more qualitative, but also more easily transferable, insight, solid-state chemists turn to concepts like ionic radius and tolerance factor, which fundamentally derive from optimizing local cation-anion coordination in terms of geometry and bond lengths (Goldschmidt, 1926; Cai et al., 2011; Mouta et al., 2013; Song and Liu, 2020). These concepts do not easily translate into describing order on the midrange scale, other than to say that it occurs to relieve “strain” in the lattice. At the same time, midrange order is challenging to attack by *ab initio* methods because of the large number of different atoms and large unit cells needed for realistic description.

A potentially useful step in understanding the “why” is understanding the “what” across different structure types and length scales. Thus, the goal of this paper is to describe the structures, defects, and order-disorder phenomena in the several classes of oxides mentioned above, linking, whenever possible, structural and thermodynamic behavior. By comparing these groups of materials, we propose some reasons why different types of defects and ordering schemes dominate in each group and suggest some areas for future research.

Specifically, we compare the occurrence and energetics of various types of defects and disorders in two groups of cubic oxides. The first group, as well as the one we spend most time on, consists of fluorite-related structures (pyrochlore, weberite, defect fluorite, and zirconolite) and perovskite and its derivatives. Both accommodate oxygen vacancies as well as cation disorder and cation vacancies. Their structures are not close-packed and relatively flexible, accommodating large cations with high coordination numbers (7–12), and some variability in oxidation state. The second group of cubic oxides consists of rocksalt-derived phases and spinels. Both are relatively dense structures based on cubic close packing of anions and they do not easily accommodate oxygen vacancies. They maintain charge balance by cation vacancies (and occasional cation interstitials) and variation in oxidation state of transition metal (TM) ions. They typically contain smaller ions with lower cation coordination numbers (4, 5, and 6) than the first group. We describe the various structures, compositions, and types of defects and disorder and their associated energetics for both groups of materials. In comparing these structures, disorder, and thermodynamics, we then turn to the why question, why does each structure type favor certain defects and not others.

## Fluorite-Derived Structures

### Pyrochlore

**FIGURE 1:**
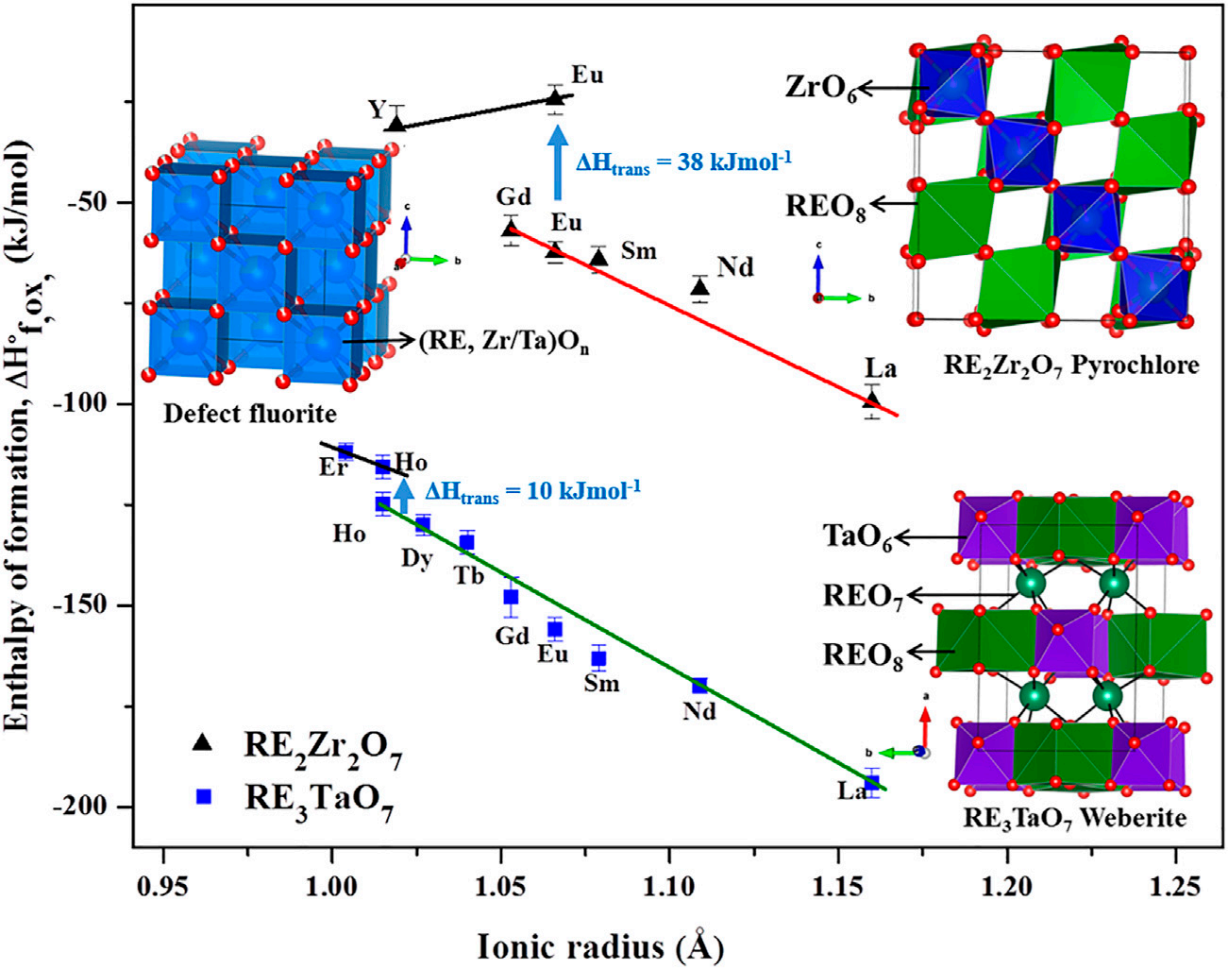
Structure and energetics of order-disorder transitions between ordered pyrochlore, weberite, and disordered defect fluorite structures with RE_2_Zr_2_O_7_ and RE_3_TaO_7_ systems as examples. The figure has been made based on data from the literature (Saradhi et al., 2012; Subramani and Navrotsky, 2019).

Pyrochlore oxides with idealized formula, A_2_B_2_O_7_, are derivatives of the fluorite structure with ordered cations and vacancies (Subramanian et al., 1983; Chakoumakos, 1984; Ewing et al., 2004; Gardner et al., 2010). While cations with diverse valences can occupy A- and B-sites, the most common composition which has been widely studied with a focus on order-disorder phenomena is A_2_B_2_O_7_ where A is a trivalent cation often rare earth and B is a tetravalent cation. The structure forms in cubic *Fd-3m* (no. 227) space group (Vanderah et al., 2005). It has five distinct Wycoff positions which are occupied by cations, anions, and vacancies. Bigger “A” cations occupy the *16d* position with eight coordinated cation coordination with oxide anions, while smaller “B” cations occupy *16c* position with six-coordinated geometry. Oxide anions go into *48f* and *8b* positions. Vacancies order in *8a* positions. Structures of defect fluorite, pyrochlore, and weberite are shown schematically in Figure 1.

The ordered pyrochlore structure disorders the defect fluorite structure under various conditions. The disordered defect fluorite structure of A_2_B_2_O_7_ is basically the fluorite structure with cubic *Fm-3m* space group but with vacancies in O sites (Minervini et al., 2000; Wuensch and Eberman, 2000). Both A and B cations are distributed in *4a* positions and both oxygen and vacancies occupy *8c* positions, giving, as a limiting ideal case, complete disorder. All A and B cations are surrounded by seven oxide ions and a vacancy (7 + 1 coordination).

The process of disordering evolves through the formation of cation antisite pairs and Frenkel defects as discussed previously (Maram et al., 2015). The various conditions that induce disorder in pyrochlore are ionic radii of A and B cations, ball milling, temperature, pressure, and radiation damage (Subramanian et al., 1983; Heremans et al., 1995; Wuensch et al., 2000; Eberman et al., 2002; Lian et al., 2003b; Liu et al., 2004; Rushton et al., 2004; Fuentes et al., 2005; Zhang et al., 2005; Zhang and Saxena, 2005; Moreno et al., 2006a; Moreno et al., 2006b; Zhang et al., 2006; Mandal et al., 2007; Mandal and Tyagi, 2007; Zhang et al., 2007; Zhang et al., 2009; Zhang F. X. et al., 2010, Zhang et al., 2010 J; Lang et al., 2010; Sanjuán et al., 2011; Sayed et al., 2011; Shlyakhtina and Shcherbakova, 2011; Zhang et al., 2015; Rittman et al., 2017b; Turner et al., 2017; Fuentes et al., 2018; Wright et al., 2021).

Helean et al. (2004) reported enthalpies of formation for RE_2_Ti_2_O_7_ systems which formed in ordered pyrochlore structure with RE = Sm - Lu. They also made similar measurements on GdTi_2_-_x_Zr_x_O_7_ solid solution (Helean et al., 2000). Lian et al. (2006) published enthalpies of formation for RE_2_Sn_2_O_7_ systems (RE = La, Nd, Sm, Eu, Dy, and Yb). The enthalpies of formation of RE_2_Zr_2_O_7_ (RE = La, Ce, Nd, Sm, and Gd) system were also reported (Navrotsky and Ushakov, 2005; Radha et al., 2009). The enthalpies of formation became less exothermic with a decrease in ionic radii from La to Lu as the structure is more prone to disorder when the tetravalent and trivalent ions are more similar in size. The measurements on the above systems were made with a focus on their behavior upon irradiation, which is important in considering them as nuclear waste forms. It was concluded that a composition more susceptible to disorder is less likely to be amorphized by radiation damage (Ewing et al., 2004).

Enthalpies of formation of hafnium pyrochlores, RE2Hf2O7 (RE = Y, La, and Gd), were reported (Lee and Navrotsky, 2004; Ushakov et al., 2007). The enthalpy of the order-disorder transition for Hf_2_Gd_2_O_7_ was 23.6 ± 3.1 kJmol^−1^ and entropy of transition was calculated as 12 J K^−1^mol^−1^, which was half of the maximum possible configurational entropy, indicating possible local ordering in the long-range disordered fluorite phase. Enthalpies of formation of CaCeTi_2_O_7_ and Ca_1.5_U_0.65_Ti_1.85_O_7_ were measured (Helean et al., 2002). Recently, calorimetric data were obtained for iridium-based pyrochlores, RE_2_Ir_2_O_7_ (RE = La, Gd, and Y) (Nenoff et al., 2021). In most of these systems, ionic radii of A or B cations play a dominant role in inducing disorder. Figure 1 shows formation enthalpies *vs.* ionic radii of different fluorite-related systems and enthalpy of an order-disorder transformation.

To understand the energetics of disordering, materials that disorder under different conditions must be studied. A disordered variant of Eu_2_Zr_2_O_7_, which usually forms in ordered pyrochlore structure, was synthesized by soft chemical and laser melt quench methods, and the thermodynamics of the order-disorder transformation were determined (Saradhi et al., 2012). The transition enthalpy from ordered to disordered phase was 37.8 ± 3.1 kJmol^−1^. *In situ* synchrotron diffraction coupled with aerodynamic levitation was performed on Eu_2_Zr_2_O_7_ to study the structural progression of disorder upon increasing temperature (Maram et al., 2015). This was followed by *in situ* synchrotron diffraction on various zirconium and hafnium-based pyrochlores, RE_2_Zr_2_O_7_ (RE = Sm, Eu, and Gd) and RE_2_Hf_2_O_7_ (RE = La, Nd, and Sm), and modeling of the thermodynamics of disordering using configurational entropy calculations based on cation and anion site occupancies obtained from Rietveld refinement (Maram et al., 2018). This study extended early work on spinel disordering to fluorite-related systems (Navrotsky and Kleppa, 1967). The enthalpies of anion Frenkel disorder were found to be smaller than those of cation antisite disorder.

Hayun et al. (2012) investigated the energetics of highly disordered RE_2_Ti_2_O_7_ (RE = Y, Gd, and Dy) formed by ball milling of their constituent binary oxides and of ordered variants formed by annealing the disordered materials at different temperatures. They found that the order-disorder transition upon annealing occurs through a two-step process.

With ionic radius, temperature, and ball milling already known to be important to induce disorder, radiation damage was then used as another “knob” to study the disorder energetics. Three different compositions in RE titanate and stannate pyrochlore systems were studied to understand the energetics of disordering induced by radiation damage (Chung et al., 2018b). Study of radiation damage, structural evolution upon annealing, and energetics of Dy_2_Ti_2_O_7_ pyrochlore used a combination of nondestructive methods [pair distribution function (PDF) analysis of neutron total scattering] followed by destructive methods (high-temperature solution calorimetry and differential scanning calorimetry). The results showed that the radiation amorphized sample had been destabilized by 243 kJmol^−1^. An important observation was that, upon annealing to 1,200°C, the amorphized sample never recovered completely structurally or energetically, with enthalpy of formation closer to that of disordered Dy_2_Ti_2_O_7_ produced by ball milling (Hayun et al., 2012) than to that of pristine undamaged Dy_2_Ti_2_O_7_, indicating a residual destabilization in the recrystallized pyrochlore. The neutron results showed that local disordered short-range domains containing a weberite-like structure (see below for discussion of weberites), present in the amorphized sample, persisted in the annealed material. The results were consistent with weberite-type short-range ordered domains observed in disordered pyrochlores (Shamblin et al., 2016a). Chung et al. (2018a) also studied the structural evolution and energetics in another pyrochlore Dy_2_Sn_2_O_7_ and found residual destabilization similar to that in Dy_2_Ti_2_O_7_. They then chose Er_2_Ti_2_O_7_ and induced disorder by ball milling and radiation damage to study the energetics (Chung et al., 2019). They again found similar retention of local short-range weberite structure and concluded that the residual destabilization was a general phenomenon in radiation-damaged pyrochlores.

Thermodynamics of the composition dependence of order-disorder in pyrochlore solid solutions has been studied in Nd_x_Zr_1-x_O_2-0.5x_ system (Finkeldei et al., 2017). They found that an order-disorder transition occurred at 0.30 < x < 0.33. Based on calorimetric data, they showed a disordering transition enthalpy of 30 kJmol^−1^and transition entropy of 16 J K^−1^mol^−1^, yet again far less than the configurational entropy, showing evidence for local ordering.

Though order-disorder transitions have been induced by pressure in pyrochlores, not much thermodynamic work has been carried out on them. New pyrochlores with A_2_B_2_O_7_ stoichiometry have been suggested to form under high pressure (Zhou and Wiebe, 2019). It will be interesting to study the energetics of pressure-induced order-disorder transformations to explore behavior under extreme conditions.

### Weberite

The weberite structure with A_2_B_2_X_7_ stoichiometry is also derived from the aristotype fluorite structure, with ordering in cation and anion sublattices. The weberite structure can include different cations with different charges in A- and B-sites and fluoride or oxide ions in the anion X sites. The structural and compositional diversity has been clearly described elsewhere (Cai and Nino, 2009; Cai and Nino, 2011). The discussion in this review will be limited to weberite oxides A_2_^3+^B_2_^4+^O_7_ and A_3_^3+^B^5+^O_7_, which are currently the only systems studied for the structural and energetic evolution of order-disorder.

Fluorite-derived oxides with cation ratio 1:1 and molecular formula, A_2_^3+^B_2_^4+^O_7_, usually form in the pyrochlore rather than the weberite structure (Subramanian et al., 1983). However, A_2_^3+^B_2_^4+^O_7_ with weberite structure was discovered by Shamblin et al. (2016a), while analyzing the local structure of A_2_B_2_O_7_ pyrochlores with disorder induced by various conditions, thus realizing that the weberite structure is a metastable form for this stoichiometry. A_3_^3+^B^5+^O_7_ phases form in the same orthorhombic space group as weberite A_2_^3+^B_2_^4+^O_7_ (Allpress and Rossell, 1979; Wakeshima et al., 2004; Cai and Nino, 2007; Cai and Nino, 2009; Fu and IJdo, 2009; Cai et al., 2010; Cai and Nino, 2011; Nakamura et al., 2011; King et al., 2013). There has been some confusion on which space group (*C2221* or *Ccmm*) best describes the structure. Gussev et al. (2020) recently showed that the space group *C2221* describes the weberite structure based on the short-range and long-range structure using neutron total scattering data and DFT calculations.

**FIGURE 2:**
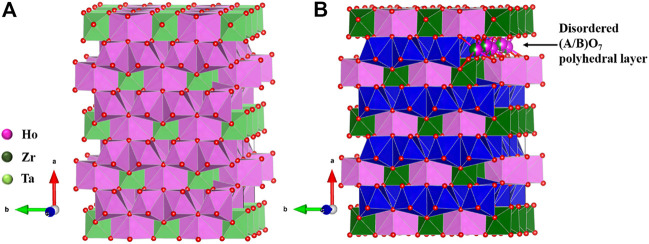
Structure of weberite-type **(A)** Ho_3_TaO_7_ and **(B)** Ho_2_Zr_2_O_7_, both forming in orthorhombic *C222*_*1*_ space group. The (A/B)O_7_ layer in which partial disorder between A and B cations is marked with blue polyhedra for clarity in **(B)**. Figure has been drawn based on the crystallographic data from the literature (Drey et al., 2020; Gussev et al., 2020). The crystal structures are drawn using the software VESTA (Momma and Izumi, 2008).

The weberite structure (Figure 2) has three distinct sites for cations, namely, *4b*, *4b*, and *8c*, and five different sites for oxide ions, *8c*, *8c*, *4a*, *4a*, and *4a*. A^3+^ cations occupy one *4b* and *8c* sites and B^5+^ cations occupy other *4b* sites in weberite-type A_3_^3+^B^5+^O_7_ oxides.

In A_2_^3+^B_2_^4+^O_7_, A^3+^ and B^4+^ go into 4b sites, and 8c sites are occupied equally by both A^3+^ and B^4+^. 4b sites form a layer of the weberite-type structure in which the A cation takes eightfold coordination forming chains of edge-shared AO_8_ cuboctahedra and B cations go into sixfold coordination forming chains of edge-shared BO_6_ octahedra along the c axis. Then, both AO_8_ and BO_6_ chains share edges in an alternating arrangement along the b axis to form AO_8_-BO_6_ layers. The difference between A_3_^3+^B^5+^O_7_ and A_2_^3+^B_2_^4+^O_7_ comes in the other layer having 8c sites which lead to sevenfold coordination with a distorted pentagonal bipyramidal geometry. The 8c sites are occupied completely by A^3+^ cations forming edge-shared AO_7_ polyhedral chains along the c axis and the chains in turn share edges along the b axis to form the AO_7_ layer. AO_8_-BO_6_ and AO_7_ layers arrange alternately to form the overall structure (Gussev et al., 2020). However, in A_2_^3+^M_2_^4+^O_7_, 8c sites are occupied equally by both A^3+^ and B^4+^ forming (A/B)O_7_ polyhedral layer leading to disorder (Shamblin et al., 2016a; Drey et al., 2020). So, the structure can be seen as an alternating arrangement of ordered and disordered layers in weberite-type, A_2_M_2_O_7_. In other words, the structure is intermediate between ideal pyrochlore (fully ordered) and defect fluorite (fully disordered), with, presumably, intermediate configurational entropy. This partially disordered weberite structure is found in the short-range ordered domains of disordered defect fluorite materials and annealed radiation-damaged materials, suggesting that a completely ordered state is difficult to achieve in these materials and may require annealing at higher temperatures for a longer time.

The thermochemistry of two systems with A_3_BO_7_ (A = RE; B = Nb, and Ta) composition has been studied. Mielewczyk-Gryn and Navrotsky (2015) reported enthalpies of formation of RE_3_NbO_7_ (RE = Y, La, Nd, Gd-Er, and Yb) with order-disorder occurring in compounds with RE ionic radii smaller than Tb. Energetics of another system, RE_3_TaO_7_ (La, Nd, Sm-Yb), was reported by Subramani and Navrotsky (2019). In both systems, enthalpies of formation became less exothermic as RE ionic radii decreased, showing a trend similar to RE_2_M_2_O_7_ pyrochlores. The occurrence of ordered and disordered variants for a single composition, Ho_3_TaO_7_, enabled the study of the energetics of the order-disorder transition. It was found that the enthalpy of transition between ordered and disordered variants was near 10 kJmol^−1^ (Figure 1). Based on configurational entropy calculations, substantial short-range ordering (SRO) in long-range disordered Ho_3_TaO_7_ was proposed.

**FIGURE 3:**
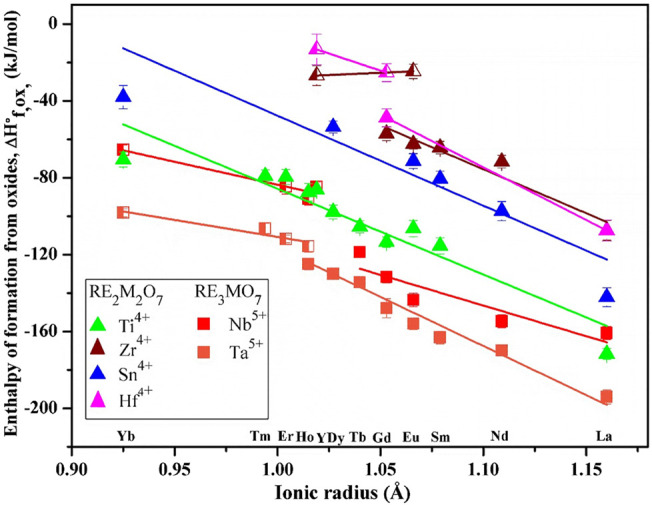
Enthalpies of formation *vs.* ionic radius of rare earth ions. The filled squares show compounds with weberite structure. The filled triangles represent compounds with ordered pyrochlore structure. The half-filled squares and triangles indicate compounds with disordered defect fluorite structure. The data for RE_2_Ti_2_O_7_ and RE_2_Sn_2_O_7_ are from Helean et al. (2004) and Lian et al. (2006). The data for RE_2_Zr_2_O_7_ are from Navrotsky and Ushakov (2005), Radha et al. (2009), and Saradhi et al. (2012). The data for RE_2_Hf_2_O_7_ are from Navrotsky and Ushakov (2005) and Ushakov et al. (2007). The data for RE_3_MO_7_ (M = Nb and Ta) are from (Mielewczyk-Gryn and Navrotsky (2015) and (Subramani and Navrotsky (2019).

Figure 3 shows enthalpies of formation *vs.* RE ionic radius. The data are based on high-temperature oxide melt solution calorimetry experiments performed by Navrotsky and coworkers over the past two decades. The plot clearly shows a trend of enthalpies of formation becoming less exothermic as RE ionic radii decrease for various fluorite-related systems. It is noteworthy that few systems have a single composition that exhibits both ordered and disordered polymorphs. A linear fit was used separately on enthalpies of formation of compositions with ordered and disordered structures in each system. The slopes show some scatter for ordered structures but are similar for ordered structures and are much less steep for disordered ones. In the future, one may be able to estimate enthalpies of formation for a whole series of rare earth by measuring the enthalpy of formation of a single composition in the system and applying the average slope.

Thermochemistry of pure weberite A_2_B_2_O_7_ materials cannot be measured directly because such phases generally do not exist. The radiation-damaged and annealed samples that Chung et al. (2018a) measured contained weberite-like short-range ordered local domains, but the exact amount of such domains is hard to estimate. The thermochemical measurements made by Hayun et al. (2012) on disordered A_2_B_2_O_7_ samples produced by ball milling might have had similar short-range ordered weberite domains, but there are no samples left for further studies. Drey et al. (2020a) described disordering in an unirradiated Ho_2_(Ti_1-x_Zr_x_)_2_O_7_ solid solution in which one of the end members, Ho_2_Ti_2_O_7_, had the ordered pyrochlore structure and other, Ho_2_Zr_2_O_7_, formed in the disordered defect fluorite structure. They investigated the long-range and short-range ordering using Rietveld refinement and PDF analysis of neutron scattering data. They found that an order-disorder transition occurred abruptly at the long-range crystallographic scale around x = 0.6, while order-disorder occurred gradually with increasing x on the short-range (nano)scale. The measured heats of mixing suggested that the solid solution could be considered a mixture of two phases at the nanoscale, agreeing with local structure studies. The structure of Ho_2_Zr_2_O_7_ was reported to be completely weberite *C2221* at the short-range scale by neutron PDF analysis. Thermochemical data for another related solid solution between pyrochlore Y_2_Ti_2_O_7_ and defect fluorite Y_3_NbO_7_ were reported recently (Winiarz et al., 2020). Though niobium-rich members formed in a disordered structure based on XRD analysis, there were additional reflections indicating possible superstructure. More structural analysis is needed to delineate these order-disorder phenomena.

Understanding the energetics of order-disorder transitions induced by other parameters like temperature, radiation damage and pressure in weberite materials are open fields for future studies. Recently, a new oxide of composition MgTiSi_2_O_7_ with weberite structure in the MgO-SiO_2_-TiO_2_ system has been discovered under high pressure and high-temperature conditions similar to the ones found in the Earth’s transition zone and lower mantle (Bindi et al., 2017; Matrosova et al., 2020). MgTiSi_2_O_7_ weberite has cation occupancy disordering with Mg and Ti ions sharing the A cation sites and Si and Ti occupying the B cation sites (Bindi et al., 2017). This may lead to some “entropy stabilization.” The work suggests the possibility of finding various new compositions with fluorite-related structure in planetary interiors. Thus, thermochemistry of pressure-induced order-disorder transitions will be interesting for pyrochlores in view of understanding their formation, existence, and role in planetary interiors.

### Other Fluorite-Related Structures

Y_2_O_3_ (YO_1.5_) doping in zirconia (ZrO_2_) introduces oxygen vacancies and leads to stabilization of zirconia in disordered cubic defect fluorite structure. These materials are called cubic yttria-stabilized zirconia (c-YSZ). The enthalpies of formation of disordered c-YSZ materials in the YO_1.5_-ZrO_2_ solid solution have been measured (Lee et al., 2003). They found that the enthalpy of mixing was negative with a very negative interaction parameter suggesting substantial SRO. Annealing produced an ordered δ-phase (Y_4_Zr_3_O_12_) and enabled the study of the energetics of order-disorder. There is no difference in the enthalpy of solution of the δ-phase and the disordered c-YSZ solid solution. This suggests strong short-range order in the nominally disordered c-YSZ. The appearance of the δ-phase superstructure in XRD patterns may indicate growth of domain size in ordered regions rather than the onset of ordering. Lee and Navrotsky (2004) reported even more negative interaction parameters for YO_1.5_-HfO_2_ solid solution than for YO_1.5_-ZrO_2_ and suggested even stronger SRO. Simoncic and Navrotsky (2007a) studied different REO_1.5_-HfO_2_ solid solutions which formed in nominally disordered structure and again found strong negative interaction parameters indicating SRO (Simoncic and Navrotsky, 2007a). Simoncic and Navrotsky (2007b) found similar negative interaction parameter for other RE_2_O_3_(REO_1.5_)-ZrO_2_ and RE_2_O_3_(REO_1.5_)-HfO_2_ solid solutions, confirming and generalizing short-range order in these long-range disordered materials as well.

The enthalpies of formation of CeO_2_ doped with La_2_O_3_, Gd_2_O_3_, and Y_2_O_3_, are positive (Chen and Navrotsky, 2006) in contrast to YO_1.5_-ZrO_2_ and YO_1.5_-HfO_2_ systems The reason for such behavior was attributed to the larger ionic radius of Ce^4+^ than that of Zr^4+^ and Hf^4+^ ions, making 7-fold coordination less favorable for the larger tetravalent ion and limiting the favorable energetics associated with oxide ion transfer (Chen et al., 2005). La_2_O_3_ and Y_2_O_3_-doped ThO_2_ were studied by Aizenshtein et al. (2010). The energetics of these two systems were balanced by competition between a destabilizing factor due to cation size mismatch and a stabilizing factor due to defect clustering. The compositions with maximum endothermic enthalpies of formation in both La and Y doped ceria and thoria systems exhibited maximum ionic conductivity. As clustering became more pronounced at higher doping levels, ionic conductivity stayed constant or decreased while the mixing energetics became more favorable. Buyukkilic et al. (2012) and Buyukkilic et al. (2014) carried out a systematic study on the energetics of singly and doubly doped CeO_2_ with Nd_2_O_3_ and Sm_2_O_3_. The doubly doped system had less average size mismatch, and the maxima in conductivity and heat of mixing were both shifted to higher doping levels, with heat of mixing smaller in magnitude. Enthalpies of defect association of the doubly doped (Nd.Sm) in a 1:1 ratio system were less exothermic than those of singly doped (Nd or Sm) systems. These studies show that ionic conductivity, defect association, and heats of mixing are closely related.

Zirconolites [CaMTi_2_O_7_ (M = Zr and Hf)] are another set of pyrochlore-related compounds derived from fluorite with defects but with lower symmetry and higher ordering for cations (Grey et al., 2003; Salamat et al., 2013; McCaugherty and Grosvenor, 2019). They are studied for the possible application as nuclear waste materials to host actinides in high-level waste (Vance et al., 1994; Ewing et al., 2004). Various types of disorder have been induced in the zirconolite structure using chemical substitution and high pressure (Whittle et al., 2012; Salamat et al., 2013). The enthalpies of formation of undoped CaMTi_2_O_7_ (M = Zr and Hf) and uranium doped CaZrTi_2_O_7_ have been reported (Putnam et al., 1999a; Putnam et al., 1999b; Subramani et al., 2020). However, energetics of disordering in zirconolites have not been explored in depth. Such studies would be very useful to understand the process of disordering in such highly ordered systems.

## Perovskite and Related Structures

### Structure and Occurrence

**FIGURE 4:**
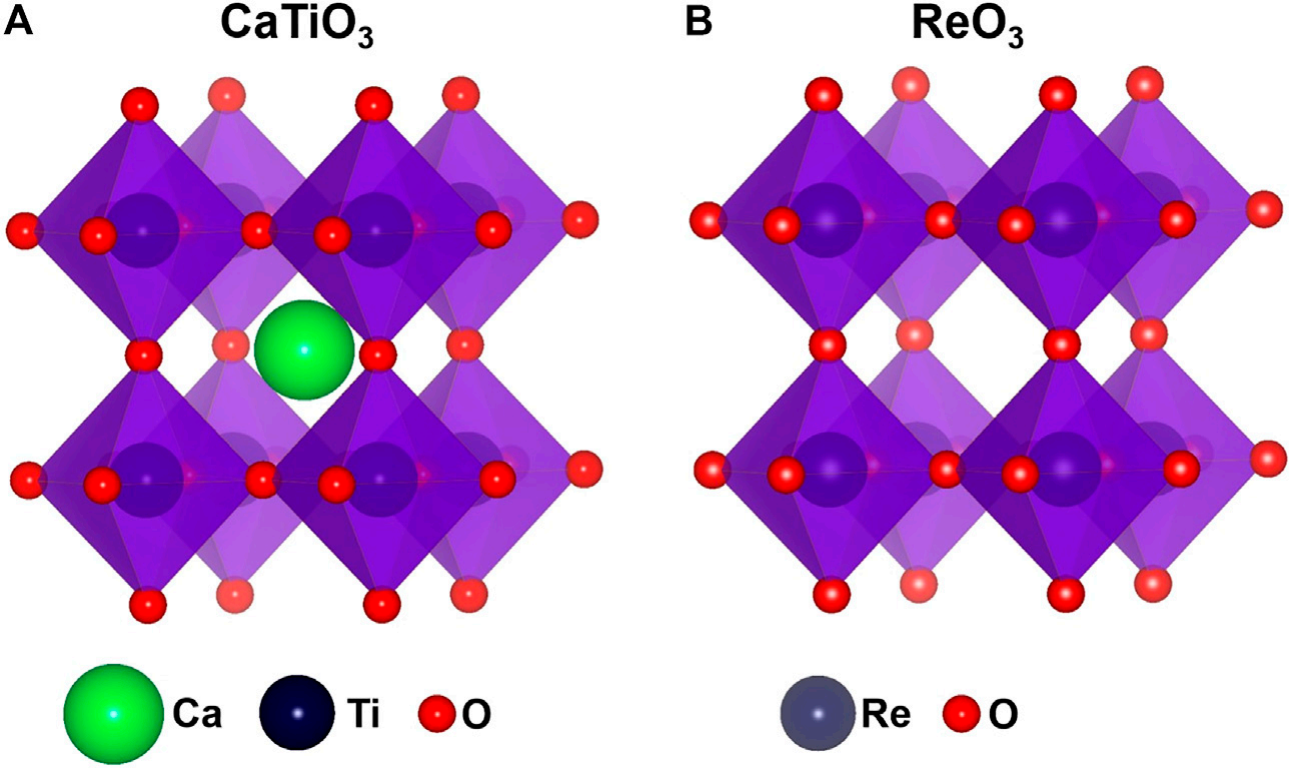
**(A)** CaTiO_3_ perovskite structure and **(B)** ReO_3_ structure with empty cuboctahedral A-site.

Perovskites derived from the ideal cubic aristotype structure are among the most, if not the most, rigorously investigated class of materials. Perovskite *sensu stricto* is a CaTiO_3_ mineral and compounds with the isotypical crystal structures and ABX_3_ (where A is an alkali, alkali earth, or lanthanide metal, B is a TM, and X is an oxygen or halogen atom) general formula are classified as inorganic perovskites. The perovskite structure is based on a corner-sharing array of octahedra which creates a large central site with room for a larger cation. This structure has flexibility in terms of rotation and distortion of the octahedra. The ideal cubic ABX_3_ perovskite (space group *Pm3m*) has a large A cation at a twelve-coordinated site (A-site) by the X anions and a medium-size B cation at a six-coordinated site (B-site). The B-site cations are strongly bonded with the X anion, while A-site cations have weaker interactions. It forms a three-dimensional network of the corner-sharing BX_6_ octahedra where A cations occupy empty cuboctahedral cavities (Figure 4A). If one removes all A cations from the twelve-coordinated sites, the simple ReO_3_ structure is generated (Figure 4B).

In perovskite oxides, the A-site cation can have +1, +2, or +3 oxidation states, whereas the B-site cation can have a +3, +4, or +5 oxidation state to realize the charge neutrality of ionic structure. The presence of oxygen excess or deficiency in the lattice can cause the departure of stoichiometry from the ideal ABO_3_. The strongly bonded framework of octahedra allows changing the elemental composition of A- and B-sites or partial substitution of cations without structural collapse. The presence of empty space in the perovskite structure can accommodate various octahedral tilts and distortions, reflecting the flexibility of the framework of octahedra. As a result, countless new perovskites with fascinating physical and chemical properties have been synthesized and have found applications in almost all areas of modern technology. Furthermore, the significance of perovskites extends to and beyond the Earth, since silicate perovskites based on MgSiO_3_ are thermodynamically stable in the mantle of planets larger than Mars (Szuromi and Grocholski, 2017).

Reflecting the large flexibility of its crystal structure and its ability to show different properties depending upon the environment, perovskite can be well described as an “inorganic chameleon” (Stølen et al., 2006). The perovskite oxides commonly crystallize in a cubic structure, but, depending on the ionic radii and electronegativity of the corresponding cations, tilting or expansion/contraction of the octahedra occurs, leading to the formation of lower symmetry structures. The empirical prediction of whether the ABX_3_ compound can form a stable cubic structure can be derived from a dimensionless number called the Goldschmidt tolerance factor *t*:

$$t=\frac{\left(r_{a\;}+\;r_{b}\right)}{\sqrt{2}\left(r_{b\;}+\;r_{x}\right)}$$

where r_a_, r_b_, and r_x_ are the ionic radii of A, B, and X ions, respectively (Goldschmidt, 1926). The closer this factor is to unity, the better the normal metal-oxygen bond lengths of both cations can be satisfied without distortions of the ideal structure. The tolerance factor possesses acceptable predictive power for oxide and fluoride structures. The ideal cubic perovskite SrTiO_3_ has t = 1, and the closer the t value to unity, the greater the energetic stability of the structure (Navrotsky and Donald, 1989; Navrotsky, 1994). If t is between 0.9 and 1.0, predominantly cubic structures form, while below and above that limit significant distortions of the structure take place. When B is large and A is small, t < 1, the structure lowers its symmetry to fill the space, whereas if t > 1, hexagonal perovskite forms with face-sharing octahedra. The thermodynamic stability of various perovskites within a large compositional range has been extensively studied by high-temperature melt solution calorimetry in Navrotsky’s laboratory (Linton et al., 1998; Laberty et al., 1999; Xu et al., 2003; Cheng et al., 2005; Cheng and Navrotsky, 2005; Xu et al., 2005).

**FIGURE 5:**
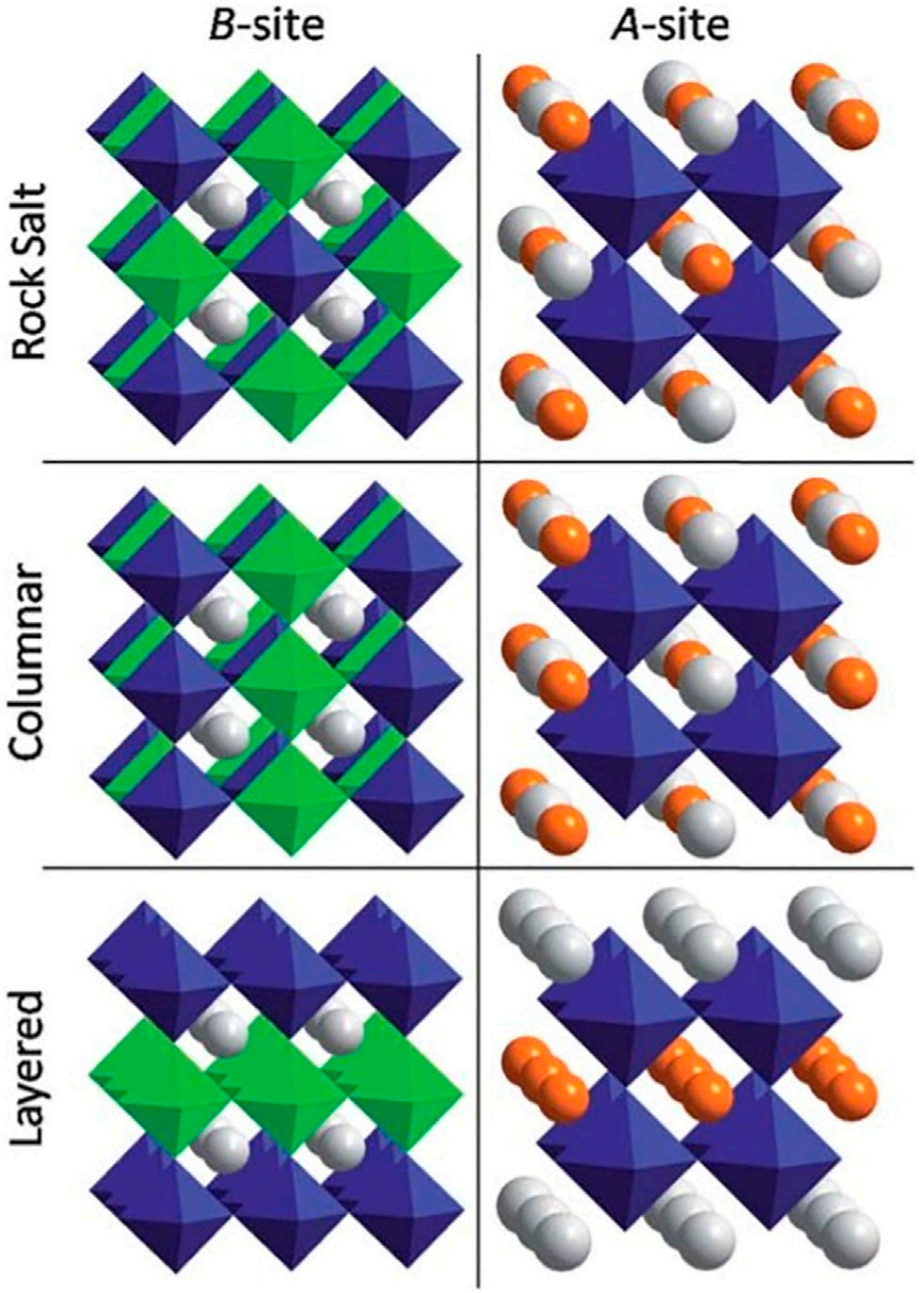
Cation ordering possibilities in perovskites for B-site ordering in A_2_BB’X_6_ and for A-site ordering in AA’B_2_X_6_ perovskites. Reproduced with permission from King and Woodward (2010), Copyright Royal Society of Chemistry.

Perovskite properties can be tuned to a great degree by partial cation substitution. It can take place at either the A- or the B-site resulting in the double perovskites with A_2_BB’X_6_ or AA’B_2_X_6_ formula, respectively. The A/A’ and B/B’ cations may remain disordered at their corresponding sites, or they can order forming A-site or B-site ordered perovskites (Smyth, 1985; Anderson et al., 1993; Davies et al., 2008; King and Woodward, 2010; Vasala and Karppinen, 2015; Tilley, 2016). Three ordering patterns can be realized for either the A- or B-site cations. The most symmetric and most frequently encountered group is called rock salt or 1:1 ordering because the pattern of two different cations is equivalent to the anion and cation positions in the NaCl-type structure with Fm3m symmetry. This superstructure is also known as the elpasolite (K_2_NaAlF_6_) structure. Besides rocksalt ordering, cations can order into columns, or layers as shown in Figure 5 (King and Woodward, 2010).

These types of ordering are less common than the rocksalt-type ordering. The NaCl-type arrangement is 0D since two compositionally similar octahedra are alternating and separated from each other. The 1D and 2D ordering patterns result from columnar or layered ordering since it allows connectivity of the B’X_6_ octahedra in one or two dimensions, respectively. The energetic stability of cation order is mainly driven by the charge and size difference of B/B’ or A/A’ cations. Among all three ordering patterns, the rocksalt-type ordering is usually thermodynamically most favorable because it increases the separation between highly charged B’ cations and hence decreases the Coulombic repulsive interactions. Then, it comes columnar configuration and 2D ordering is the least favorable due to the presence of four B’ cations neighboring with another B’ cation. Depending on the stoichiometric ratio of B:B’, the 1:2 and 1:3-layer ordering of cations can take place, which is the closest analog to the NaCl-type ordering (Davies et al., 2008). A-site ordering in perovskites is much less common compared to the B-site ordering. One way of achieving A-site ordering is by octahedral tilting which creates different size voids within the interconnected BX_3_ network. The a^+^a^+^a^+^ tilting stabilization is the most commonly occurring type among the Glazer tilt systems (King and Woodward, 2010). The perovskites with this type of tilting usually contain larger cations such as an alkali, alkaline earth, or rare earth at the A-site, with much smaller cation at the A’-site. Cubic perovskites with AA_3_’B_4_O_12_ general formula accommodate this type of cation ordering and compounds with this structure can form a metastable high pressure ζ-Mn_2_O_3_ phase (Ovsyannikov et al., 2013; Tilley, 2016). However, the number of reported A-site ordered perovskites is significantly smaller than the B-site ordered ones, and therefore the interesting question is why B-site ordering is more common than A-site ordering. This can be due to the small charge or size mismatch between A and A’ cations, while the difference of cation sizes and charges between B and B’ atoms can be larger. Generally, a large difference in valence, size, and coordination between two cations located at the same crystallographic site leads to the stabilization of a more ordered structure.

### Oxygen Vacancy Formation

Another uniqueness of perovskite relative to many other crystal structures is its ability to accommodate a large concentration of oxygen vacancies (ABO_3-δ_) without structural decomposition. The most oxygen deficiency is achieved close to δ = 0.5, although even nonstoichiometry higher than δ = 0.8 has been reported for Ba_1-x_Sr_x_Co_1-y_Fe_y_O_3-δ_ composition (Kubicek et al., 2017).

The ordering of oxygen vacancies along the (110) direction relative to the perovskite structure at δ= 0.5 often results in a new family of compounds known as brownmillerite (Ca_2_AlFeO_5_) with a layering of differently coordinated B and B’ cations (Smyth, 1985; King and Woodward, 2010; Tilley, 2016). The oxygen-deficient octahedra then transform into tetrahedra forming alternate layers of BO_6_ octahedra and BO_4_ tetrahedra in the … OTOT … sequence. The room temperature stable ordered brownmillerite transforms into perovskite with disordered oxygen vacancies at elevated temperatures (>800°C) (Shin et al., 1978). The transformation is gradual involving the coexistence of intermediate ordered and disordered phases or it may appear first order (Prasanna and Navrotsky, 1994). Disordering occurs through the formation of defect clusters which are statistically distributed in the cubic perovskite framework. Like in perovskites, B-site ordering is quite common for B-site substituted brownmillerites (King and Woodward, 2010). Also, the concentration of defects can be significantly altered through doping with different cations, often resulting in disordered cubic phases (Patrakeev et al., 2006). Another important parameter that affects the composition and phase formation is the oxygen partial pressure during the synthesis. If variable valence cations such as iron are present, low oxygen partial pressures brownmillerite and disordered perovskite phases can coexist, while at elevated temperatures transformation into disordered or ordered cubic structure takes place (Prado et al., 2004). The addition of oxygen to a brownmillerite structure can convert some of the tetrahedral layers into octahedral on forming intermediate intergrowth phases (A_n_B_n_O_3n-1_) between pure perovskite and brownmillerite. For example, at *n* = 3, Grenier compounds with … OOTOOT … stacking can be generated. The whole range of oxygen-deficient intermediate compounds 0 < δ < 0.5 may be thermodynamically metastable relative to the stable perovskite and brownmillerite end members. Indeed, it has been shown that Ca_3_Fe_2_TiO_8_ and Ca_4_Fe_2_Ti_2_O_11_ intermediate phases are energetically metastable relative to their parent CaTiO_3_ and Ca_2_Fe_2_O_5_ (Prasanna and Navrotsky, 1994).

Square pyramidal BO_5_ coordination polyhedra can form if the oxygen vacancies are ordered in single octahedral sites rather than in two sites as in brownmillerite. These types of compounds include manganites (e.g., Sr_2_Mn_2_O_5_), cobaltites (e.g., LaBaCo_2_O_5.5_), and ferrites (e.g., SrFeO_2.5+δ_) (Tofield et al., 1975; Caignaert et al., 1985; Rautama et al., 2009). Another interesting family of oxygen-deficient perovskite is cuprates ACuO_3-δ_ in which copper atoms have square pyramidal and square planar coordination (Murphy et al., 1987; Rao and Raveu, 1989). Due to such unique structural geometry, these materials (e.g., YBa_2_Cu_3_O_x_) exhibit high-temperature superconducting properties (Murphy et al., 1987).

Creating A-site deficiency, while retaining the corner-shared octahedral framework, can be realized in the so-called perovskite tungsten bronzes A_x_WO_3_, which undergo multiple structural changes upon increasing the concentration of A cations. For example, orthorhombic Ca_0.01_WO_3_ transforms into tetragonal phase at Ca_0.03_WO_3_ and eventually into cubic at Ca_0.12_WO_3_ (Tilley, 2016). Titanates, tantalates, and niobates are also capable of withstanding partially occupied A-sites if the charge neutrality is preserved (e.g., Ce_0.33_NbO_3_).

If the A-sites are completely empty in the perovskite structure and B cation has +6 oxidation state, cubic ReO_3_ structure forms (Figure 4B). The slight reduction of anion content in some oxides with ReO_3_ structure generates randomly distributed point defects, which undergo ordering and subsequent annihilation at high concentration of anion vacancies forming planar 2D defects known as crystallographic shear (CS) planes (Magneli, 1956; Wadsley, 1958; Voskanyan and Navrotsky, 2021). This suggests that the ordering of defects is thermodynamically favorable. Recently, Voskanyan and Navrotsky showed that TiO_2_-Nb_2_O_5_ Wadsley-Roth CS phases are energetically metastable and stabilized *via* configurational entropy arising from the cation disorder at elevated temperatures (Voskanyan et al., 2020). In many oxides, defect clustering at the short-range scale is energetically more favorable and long-range ordering of vacancies does not take place. The reason for this is still not well understood and needs further theoretical and experimental investigations. In particular, the distribution and optimization of cluster size need further study.

Although some of the ReO_3_ based compounds form CS phases, perovskites with occupied A-sites generally do not. This clearly indicates that the presence of filled A-sites hinders the formation of shear planes and at oxygen-deficient conditions, generation of BO_4_ or BO_5_ coordination polyhedra is more favorable in stabilizing the structure. From a kinetic viewpoint, shear plane formation involves cooperative migration of defects, which may be hampered in the presence of A cations.

Besides their high tolerance to oxygen deficiency, perovskites can also accommodate extra oxygen in their structure resulting in homologous series with A_n_B_n_O_3n+2_ general formula (Nanot et al., 1974; Smyth, 1985). For example, with the addition of extra oxygen, SrTaO_3_ perovskite can be converted into Sr_2_Ta_2_O_7_ (*n* = 4) compound which belongs to the orthorhombic crystal system with space group Cmcm (Fu and Skrabalak, 2017). It consists of perovskite-type slabs four TaO_6_ octahedra thick and two Sr sites, one within the slabs and the other between the slabs. The excess oxygen terminates one of the separated corners of the octahedra. Compared with perovskites, these compounds are likely to be also energetically metastable, and it will be of great interest to calculate the energetic penalty to form these structures from perovskites and how their energetics is compared with those of perovskite to brownmillerite transition.

## Rocksalt Structures

### Structures and Occurrence

**FIGURE 6:**
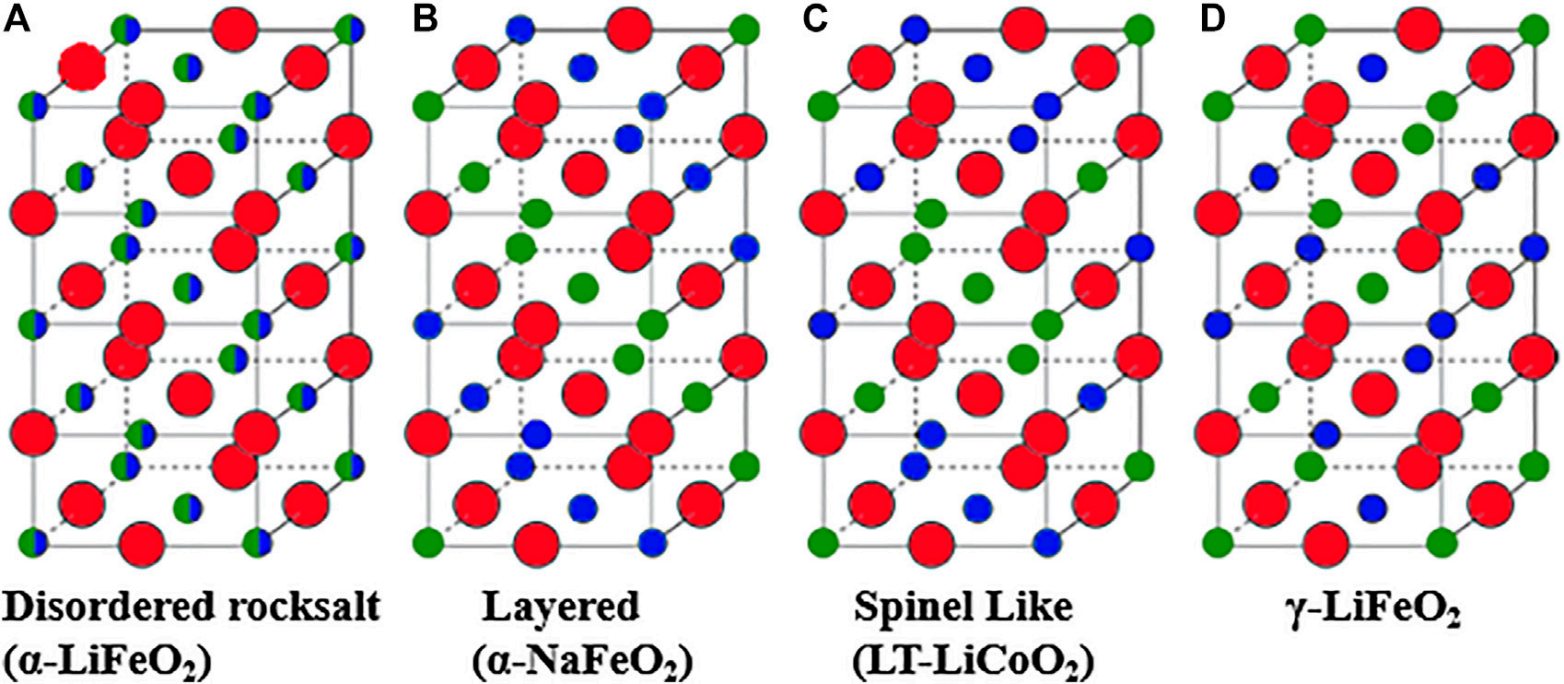
Rocksalt lithium transition metal oxides: **(A)** disordered rocksalt structure with equivalent cation sites, **(B)** layered structure with α-NaFeO_2_ structure, **(C)** low-temperature spinel-like LiCoO_2_ structure, and **(D)** γ-LiFeO_2_ structure. Oxygen is indicated by red circles and Li and TM are indicated by small green and blue circles. Adapted with permission from Urban et al. (2014), Copyright Wiley-VCH.

Lithium TM oxides (LTMO) with rocksalt structure are formed when lithium and TM occupy the cation site and oxide ion occupy anion sites of structures derived from the simple rocksalt (NaCl) aristotype. The unique feature of this rocksalt structure is that both cation and anions occupy regular octahedral coordination. Close-packed oxygen atoms occupy sites of face-centered cubic (FCC) lattices; the lithium and TM occupy the FCC sublattice of octahedral interstices. The typical cation arrangement in LTMO structures was reviewed and later explained using atomistic modeling (Hewston and Chamberland, 1987; Wu et al., 1998). The arrangement of Li and TM in these LTMO structures may vary as shown in Figure 6 (Urban et al., 2014). Disordered rocksalt oxides (DRO) exhibit a random arrangement of Li and TM (at least at the long-range scale), leading to the α-LiFeO_2_ structure shown in Figure 6A, while layered compounds exhibit an ordered arrangement of Li and TM in alternating planes along the (*111*) direction, leading to the α-NaFeO_2_ structure shown in Figure 6B. The spinel-like low-temperature structure of LiCoO_2_ and the γ-LiFeO_2_ structure are two other cation-ordered variants of the rocksalt structure shown in Figures 6C,D (Clément et al., 2020).

DRS compounds can be composed of a wide variety of TM species, Ti, Cr, Mn Fe, Nb, and Mo with a large range of compositions in this structure space, whereas the layered LTMO appear to be restricted to Ni, Co, and Mn. However, there are extensive studies on the layered structure and its stability, which is attributed to its ability to independently relax the oxygen octahedra around each type of cation (Wu et al., 1998).

The electronic, optical, and magnetic properties exhibited by LTMO are governed by the type of Li-TM bond formed which in turn are dependent on the electronic configuration and size of the trivalent cation. Hewston and Chamberland (1987) investigated first-row TM (M) LMTO and showed that partially filled t_2g_ orbitals and t_2g_-t_2g_ orbital overlap result in d electron delocalization in LiTiO_2_ and Li_x_VO_2_ resulting in paramagnetic behavior of the former and semiconducting to metallic behavior of the latter. LiScO_2_ with no d valence electrons is insulating, whereas the TM to the right of vanadium are characterized by contracted t_2g_ orbitals, prohibiting direct t_2g_-t_2g_ orbital interactions and hence exhibiting localized *d* orbitals and semiconducting behavior.

Layered rocksalt structures form when the M^3+^ cation is significantly smaller than Li^+^, as observed in LiVO_2_, LiCrO_2_, (Rudorff and Becker, 1954), LiNiO_2_, (Dyer et al., 1954), and LiCoO_2_ (Orman and Wiseman, 1984); the structure is further stabilized by independent relaxation of bond length in LiO_6_ and TMO_6_ octahedra (Wu et al., 1998). However, in LiScO_2_ (Wu et al., 1998), cations are of similar size, the relaxation effect is less significant, and the structure is dictated by electrostatic interactions resulting in γ- LiFeO_2_ structure (Figure 6D). LiMnO_2_ crystallizes in a low-temperature orthorhombic form with ordered Li^+^ and Mn^3+^ ions, unique among the other LTMO, and attributed this structure to the presence of Jahn-Teller distorted Mn^3+^ ion. However, at high temperature, the cation-ordered rocksalt structure forms (Dunitz and Orgel, 1957; Goodenough, 1959; Hoppe et al., 1975). LiCoO_2_ adopts a spinel structure with cations in 16*c* and 16*d* octahedral sites when made by low-temperature synthesis routes (Gummow and Thackeray, 1992; Rossen et al., 1993; Lee et al., 2016). Although various combinations of TM can form the layered structure, only a few will remain layered when a significant amount of Li^+^ is removed from the structure. Only those TM which not only possess very high octahedral site preference but also retain such preference on oxidation as in Ni^3+^/Ni^4+^ (d^7^ to d^6^) and Co^3+^/Co^4+^ (d^6^ to d^5^) will have high enough thermodynamic and kinetic barriers to prevent disordering of the layered structure (Clément et al., 2020).

The cation disordered structure is the high-temperature and high-entropy form of the other structure types. Disorder in rocksalt structures can also be achieved, probably metastable, by ball milling (Dachille and Roy, 1964; Gutman, 1994).

First-principles computational studies are crucial in screening the compositional space for likely disordered structure and to shed light on the origin of cation disorder (Urban et al., 2017). For metallic alloys, Hume-Rothery and Powell (1935) rules predict that alloys are formed when species have similar electronegativities and difference in atomic radii is no more than 15%, and this rule is a possible guideline for ionic materials like oxides. Disorder in LMTO occurs with TM having larger ionic radius and charge differences, while current understanding would predict increasing disorder with smaller differences. The ability of the disordered structures to accommodate distortions in their octahedra may be a stabilizing factor.

Urban et al. (2017) suggested that the ability of the structure to accommodate disorder depends on the d- orbital occupancy in the TM. When cations are randomly distributed in the rocksalt structure, differences in ionic radii and charges create distortion in the O_h_ sites; these distortions must be shared by the neighboring octahedra. Thus, the energy benefit of distorting the octahedra to better fit various cations may determine the ability of the structure to accommodate cation disorder (Clément et al., 2020). Urban et al. (2017) have investigated the octahedral distortion modes in LTMO and concluded that the TM with d^0^ electrons accommodates octahedral distortion at the lowest energy. The band energy of d^0^ TM depends on the lower-lying oxygen-dominated orbitals that are always occupied and are insensitive to site distortions (Clément et al., 2020). Disorder in the rocksalt structures is best stabilized by TM having no valence d electron even when the ionic radius and charge differences are relatively large (Urban et al., 2017). These electronic structure studies show that the redox-inactive d^0^ metals (Ti^4+^, V^5+^, Nb^5+^, and Mo^6+^) in LTMO occupy the distorted O_*h*_ site, leaving the less distorted cation site for the other redox-active TM, thus stabilizing the disordered rocksalt structure. In other words, not only does the d^0^ cation have a low-energy penalty in distorted sites, but also their flexibility to distort allows the other TM cations with d electrons to optimize their distortions. Nevertheless, some DRO with no d^0^ TM have been synthesized (Freire et al., 2017; Freire et al., 2018; House et al., 2018). Li_2_MnO_2_F possesses a disordered rocksalt structure with cation vacancies (House et al., 2018), and Li_2_MnO_3_-disordered rocksalt with vacancies enhances the electrochemical properties of cathode materials (Freire et al., 2017; Freire et al., 2018).

Murphy et al. (1982) studied Li insertion in various close-packed titanate framework. LiTiO_2_ spinel transforms to Li_2_Ti_2_O_4_ on lithiation, which is accompanied by displacement of Li from Td sites to the O_h_ sites which are vacant in the spinel structure (*Fd*-*3m* space group) resulting in rocksalt structure (*Fm-3m*). Metastable Li_2_Ti_2_O_4_ irreversibly transforms into the disordered rocksalt structure above 600°C (Murphy et al., 1982). The stable high-temperature form for LiTiO_2_ is a disordered rocksalt structure. Hua et al. (2019) had investigated structural insights in the formation of Li- and Mn-rich layered oxides; *in situ* high-temperature synchrotron radiation diffraction reveals the transformation of the lithium-rich layered phase (*R*-3*m*) to a lithium poor spinel phase (*Fd*-3*m*) *via* an intermediate lithium-containing rocksalt phase (*Fm*-3*m*) which is accompanied by the release of oxygen and lithium.

### Consequences of Cation Disorder

Ordered and disordered LMTO are cathode materials and changes in their chemical composition and structure affect the stability, capacity, energy density, and performance. Disordering results in radically different properties compared to the ordered rocksalt structure. Having excess lithium in the structure, diffusion processes are altered, easier anion redox, smaller and isotropic lattice expansion, and possible fluorine substitution, all leading to enhancement of electrochemical performance in lithium-ion battery applications.

**FIGURE 7:**
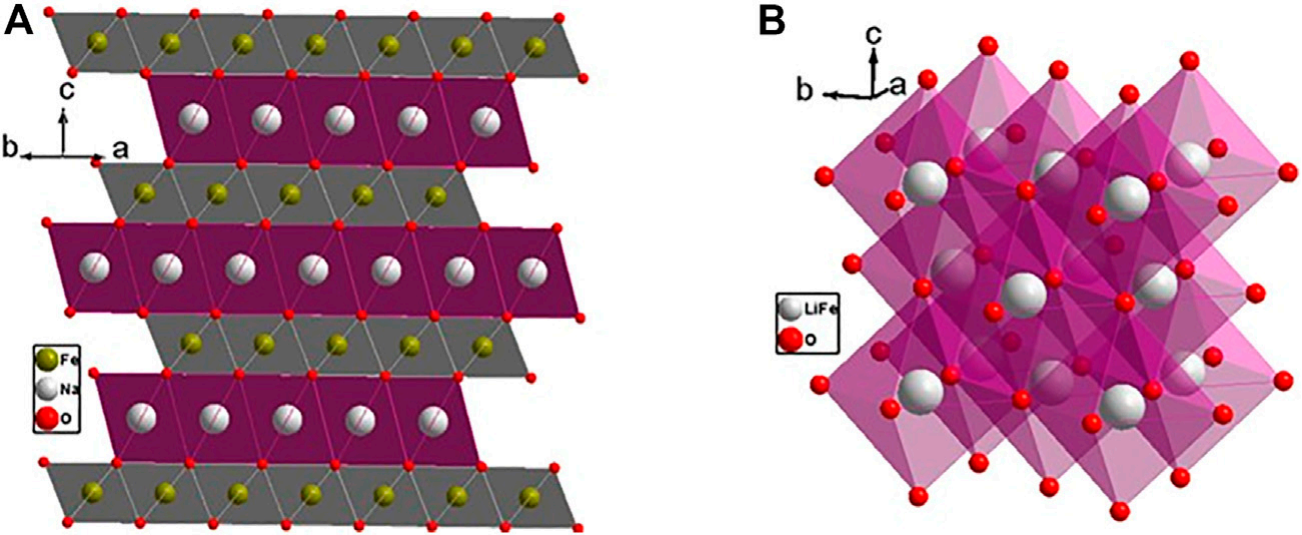
**(A)** Layered cation-ordered lithium transition metal oxide (*R*-3*m* space group); **(B)** cation disordered rocksalt structure (*Fm*-3*m* space group).

LMTO cathode materials function by reversible extraction and insertion of Li^+^. To support continuous ion migration, the cathode material has to meet the following requirements: 1) the material must have facile ion diffusion *via* low-barrier channels and 2) the diffusion channels need to form a percolating network. The diffusion mechanism involves Li^+^ ions moving from the octahedral site to an adjacent edge-shared octahedral site through the empty face-shared tetrahedral site, referred to as o-t-o diffusion. The size of the T_d_ site and electrostatic interaction between Li^+^ in the activated T_d_ site and the four cations in the face-sharing octahedra forming a tetrahedral cluster has a major impact on the Li^+^ diffusion barrier. There are five types of tetrahedral cluster formed when the Li^+^ and TM species occupy the cation lattice: 0-TM, 1-TM, 2-TM, 3-TM, and 4-TM (Kang and Ceder, 2006; Kang et al., 2006). Li diffusion pathway requires at least two O_h_ Li connected *via* an activated T_d_ site; thus, 3-TM and 4-TM environments are excluded. In layered LTMO, every T_d_ site is coordinated by either 3 Li and 1-TM or 1 Li and 3 TM, out of which only 1 TM sustains Li migration. The size of the T_d_ site is dependent on the layers’ spacing of the Li slab (Kang and Ceder, 2006; Kang et al., 2006). In layered LMTO which has a separate Li layer and TM layer (Figure 7A), the ionic radius of Li is larger than that of TM, and the layer spacing of the Li slab lies in the range of 2.6–2.7 Å (Urban et al., 2014). All the Li sites are equivalent in layered LMTO and hence all are interconnected by 1-TM and form a 2D percolating network inside the Li slab. A migration barrier is associated with 1-TM diffusion during charging because of the increasing electrostatic repulsion when the TM is oxidized to higher valence and also due to a change in the slab distance. The electrostatic attraction between the Li and the oxygen atom decreases during charging. At the end of the charge when almost all the Li would be extracted and the Li slab collapses, the lithium mobility decreases.

In cation disordered rocksalt structures, there are no separate layers for Li and TM and no defined slab distance (Figure 7B). Instead, there is a significant reduction in the slab spacing to 2.3–2.4 Å and the size of the T_d_ site due to cation mixing. The 2-TM channel is inactive and the 1-TM channel can support Li migration in cation disordered structures or if only 0-TM channels are available. In 0-TM, the absence of TM in face-sharing octahedra reduces electrostatic repulsion in the activated Li site, and the diffusion barrier is independent of the T_d_ height and the TM composition (Urban et al., 2014).

Long-range lithium diffusion depends on both the low-energy diffusion barrier and the percolating network formed by interconnecting the diffusion channel. In layered LMTO, all the Li ions are connected *via* 1-TM forming a 2D percolating network for Li conduction in Li slabs. Urban et al. (2014) have done Monte Carlo simulations to study the impact of cation disorder and Li content on Li percolation *via* the 0-TM channel. Though the amount of lithium is greater in the cation disordered structure, the percolation network of the 0-TM channel still forms at a critical Li concentration.

### SRO in Defect Rocksalt Oxides

Nominally disordered rocksalt structures do exhibit some degree of SRO. SRO is known to control local transport (Ji et al., 2019). The SRO has previously been studied by a combination of experimental (electron diffraction, solid-state nuclear magnetic resonance, and 2D and 3D nanoscale X-ray spectro-microscopy), as well as by theoretical methods (DFT and MD simulations) (Clément et al., 2018; Kan et al., 2018; Ji et al., 2019; Jones et al., 2019).

Kan et al. (2018) proposed that local cation ordering redirects Li movement through nonequilibrium pathways, which results in the chemical heterogeneity evidenced by 2D and 3D maps of manganese valence state in Li_x_Nb_0.3_Mn_04_O_2_. Ji et al. (2019) showed that SRO controls the Li transport by altering the distribution of 0-TM, 1-TM, and 2-TM channels as well as connectivity between them. Ji et al. (2019) explained the difference in the performance of Li_1.2_Mn_0.4_Ti_0.4_O_2_ and Li_1.2_Mn_0.4_Zr_0.4_O_2_ based on differences in SRO. Ji et al. (2019) had modeled SRO in a variety of Li_1.2_M′_a_M″_b_O_2_ structures (M′_a_ = V^3+^, Mn^2+^, Mn^3+^, Co^2+^, and Ni^2+^ and M″_b_ = Ti^4+^, Zr^4+^, Nb^5+^, and Mo^6+^) using DFT and rationalized SRO based on charge and size effects.

## Spinels

### Structure and Cation Distribution

The term “spinel” originates from the name of the naturally occurring mineral MgAl_2_O_4_, which is also well-known as a gemstone because of its variable color characteristics owing to the TM impurities (Malíčková et al., 2021). The spinel structural family consists of numerous materials with the basic formula AB_2_X_4_ where cation A can be either an alkali, alkaline earth, or a TM and cation B can be almost any TM or aluminum, gallium, and indium. The anion X is represented by O^2−^, S^2−^, Se^2−^, Te^2−^, F^−^, or CN^−^ species (Zhao et al., 2017). Fortunate conjunction of mixed valence, site preference, a remarkable variety of chemical compositions, and synthetic flexibility in spinels give rise to a wide range of useful functions such as magnetism (Gorter, 1950; Song and Zhang, 2004), catalytic activity (Dong et al., 2019; Kim et al., 2020), and superconductivity (Hagino et al., 1995; Moshopoulou, 2004) as well as electronic, optical, and electrochemical performance (Verwey et al., 1947; Ferg et al., 1994; Aizawa et al., 2002; Jouini et al., 2007; Kim et al., 2014).

**FIGURE 8:**
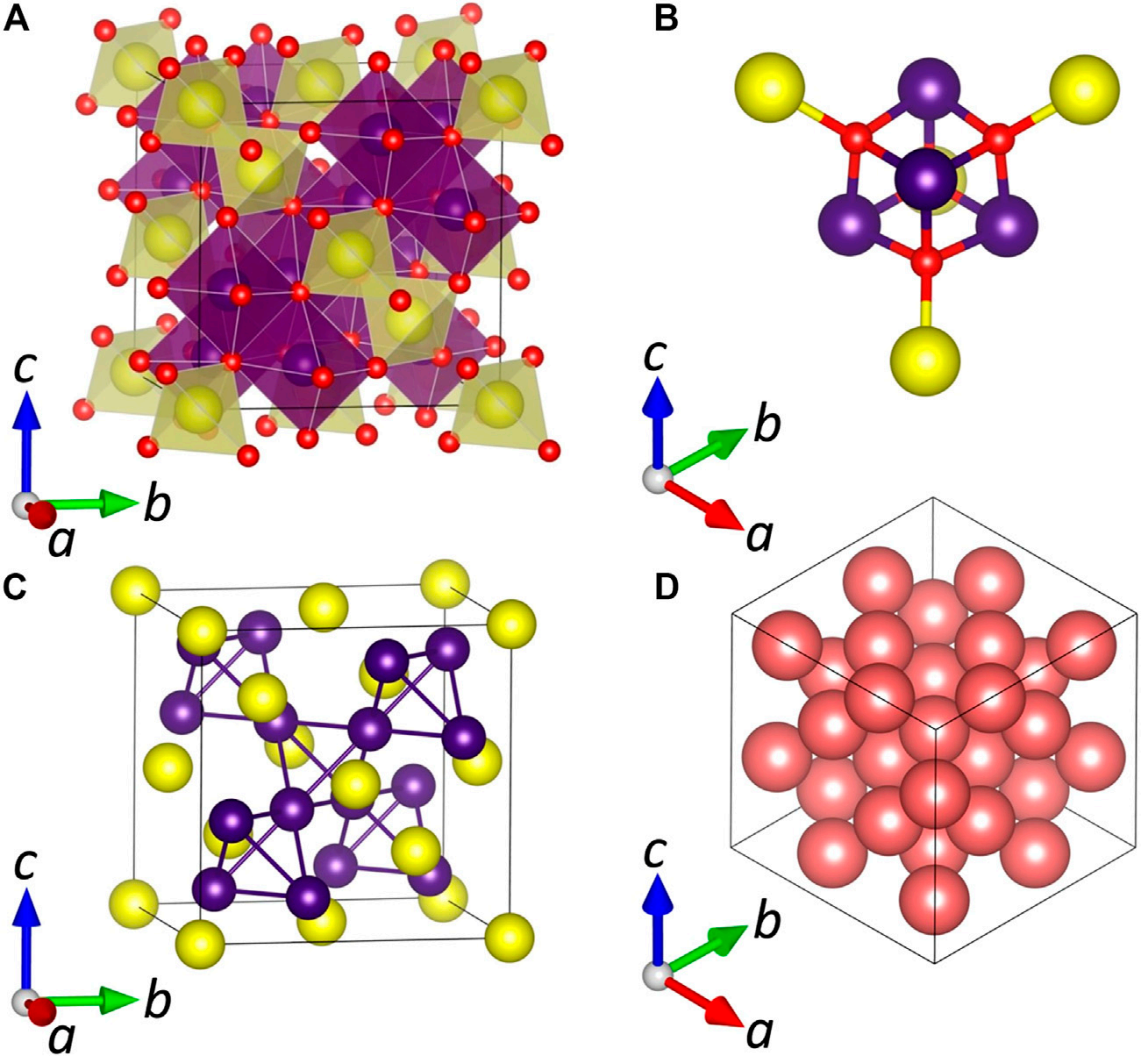
**(A)** A general view of the normal spinel unit cell ($Fd\bar{3}m$) with an origin on the A-site cation (Wyckoff site 8*a*). **(B)** A fragment of the crystal structure illustrating coordination surroundings of the X anion. **(C)** The metal sublattice AB_2_ forming a framework of the Laves phase. **(D)** A cubic close-packed array of anions in the X sublattice. A, B, and X sites are shown as yellow, purple, and red spheres, respectively.

The rich story of spinel structural chemistry began in 1915 when the crystal structure was determined for the first time in two independent works by Bragg and Nishikawa (Bragg, 1915; Nishikawa, 1915). At first glance, the structure is represented by a three-dimensional ensemble made of vertice/edge-sharing AX_4_ tetrahedra and BX_6_ octahedra (Figure 8A). A FCC unit cell ($Fd\bar{3}m$) accommodates 8 A, 16 B, and 32 X atoms resulting in eight formula units (*Z* = 8). Each anion X is tetrahedrally coordinated by one cation A situated in the tetrahedral cage and three cations B situated in octahedral cages (Figure 8B). A closer look at the unit cell reveals other important structural features. Specifically, the A sublattice possesses a diamond-like structure with a periodicity equal to the unit cell parameter. At the same time, both A and B sublattices together form a structure reminiscent of that in the Laves phase MgCu_2_ (Stein et al., 2004). Thus, the overall spinel lattice can be imagined as a Laves phase structured AB_2_ atomic array (Figure 8C) embedded into a matrix of a cubic close-packed (ccp) arrangement of X anions (Figure 8D). The ccp arrangement contains 96 available interstices in total (64 tetrahedral + 32 octahedral); however, only a fraction of them is populated by cations in the spinel structure. However, the ccp packing is imperfect, and the corresponding X layers are corrugated due to minor deviations of anion coordinates from the ideal position. From the crystallography viewpoint, these distortions originate from the flexibility of the Wyckoff site *32e*; hence an additional parameter is required to describe atomic coordinates. It is denoted as the anion position parameter *u* (also known as the oxygen parameter in oxide spinels). Depending on the choice of origin, an ideal value of *u* for the perfect ccp arrangement can be either 0.250 or 0.375 for the origin at an octahedral vacancy ($\bar{3}m$) or an A-site cation ($\bar{4}3m$, such as in Figure 8), respectively. From geometric considerations, the anion position parameter is connected to both the cubic unit cell parameter *a* and cation-ligand distances in the tetrahedral (*R*_A-X_) and octahedral (*R*_B-X_) cages according to $R_{A-X}=\surd 3a\left(u-\frac{1}{4}\right)$ and $R_{B-X}=a\sqrt{2{\left(u-\frac{3}{8}\right)}^{2}+{\left(\frac{5}{8}-u\right)}^{2}}$, or $R_{A-X}=\surd 3a\left(u-\frac{1}{8}\right)$ and $R_{B-X}=a\sqrt{2{\left(u-\frac{1}{4}\right)}^{2}+{\left(\frac{1}{2}-u\right)}^{2}}$ for the $\bar{4}3m$ and $\bar{3}m$ origin settings, respectively. Therefore, distortions in the ccp arrangement of X result in corresponding changes in AX_4_ and BX_6_ polyhedra as the tetrahedra expand while the octahedra contract with increasing *u*. This optimization of tetrahedral and octahedral bond lengths to best fit the cations in the structure is the physical reason for the deviation from ideal cubic close packing.

Figure 8 illustrates an ideal (normal) scenario in which distinct A and B cations are entirely separated in the tetrahedral and octahedral interstices; hence the chemical formula can be written as A(B)_2_X_4_ where brackets denote the octahedral site. However, as was initially shown in 1935 by Barth and Posnjak (1932), spinels may possess an inverse atomic layout in which the tetrahedral sites are fully occupied by B cations, while half of the octahedral sites are occupied by A cations and half by B cations so that the chemical formula is B(AB)X_4_. Later, the terms “normal” and “inverse” spinel were proposed by Verwey and Heilmann (1947) to denote these two limiting scenarios. In some materials, the cation distribution is very close to the normal or inverse limits. For example, the naturally occurring mineral spinel, MgAl_2_O_4_ (which gives its name to the structure), is a nearly perfect normal spinel, while Fe(NiFe)O_4_ is an example of the inverse structure (Zhao et al., 2017). The terms “normal” and “inverse” are also applicable to binary phases, in which the same TM cations with different oxidation states tend to occupy distinct crystallographic sites. As such, the mineral hausmannite (Mn_3_O_4_) has a normal spinel structure where Mn^2+^ cations occupy tetrahedral interstices, and Mn^3+^ cations occupy octahedral interstices; therefore, an accurate formula is Mn^2+^(Mn^3+^)_2_O_4_ (Garcês Gonçalves Jr. et al., 2018). In contrast, the minerals magnetite (Fe_3_O_4_) and greigite (Fe_3_S_4_) possess an inverse structure Fe^3+^(Fe^3+^Fe^2+^)X_4_ (Chang et al., 2008).

In many spinel materials, the cation distribution falls between the normal and inverse limits, so different cation species are disordered and present in both types of sites. Taking such disorder into account, the general formula of spinel has to be written as A_1-*i*_(B_2-*i*_A_*i*_)X_4_, where *i* is a degree of inversion (the inversion parameter). Although it is clear that the normal spinel (*i* = 0) corresponds to the most ordered state, the inverse and intermediate distributions possess configurational entropy. If one assumes that the cations on each sublattice are randomly distributed, the composition of maximum entropy occurs at i = 0.667, and this may be thought of as the limiting case for disordering, with both initially normal and initially inverse spinels tending toward this random distribution at high temperature.

This assumption of randomness on each sublattice permits the construction of relatively simple thermodynamic models of cation disordering and the calculation of cation interchange energies and site preference energies. Understanding the long-range cation disorder in spinels is crucial because it substantially impacts physical properties. For example, Zhu et al. (2018) performed synthesis at variable temperatures to control the crystallinity and cation distribution in the photoanode ZnFe_2_O_4_, which can be used in the water-splitting reaction. ZnFe_2_O_4_ nanorods with a higher degree of disorder have enhanced charge carrier transport and higher photogenerated charge separation efficiency. Ndione et al. (2014) established a relation between cation disorder and conductivity in the ZnCo_2_O_4_ (normal) and NiCo_2_O_4_ (inverse) spinels, where increasing disorder leads to ZnO_h_ antisite defects and increase of the *p*-type conductivity for *i* < 0.5 in the former and a metal-insulator transition for 0.5 < *i* < 1.0 in the latter. The long-range cation disorder also influences principal magnetic properties in spinels, such as magnetic transition temperature, saturation magnetization, and magnetic exchange interactions (Willard et al., 1999).

### Short-Range Order

The above considerations appear adequate for order/disorder which can be detected by commonly used techniques such as X-ray diffraction. However, there are also scenarios of the local (short-range) cation ordering that can be observed in the spinel structure. Ivanov et al. (2010) performed a study of NiFe_2_O_4_ single crystals *via* polarization Raman measurements in conjunction with lattice dynamics calculations. The number of observed Raman modes is significantly larger than one expected from the inverse spinel structure Fe(NiFe)O_4_ in which Ni^2+^ and Fe^3+^ cations are mixed statistically in the octahedral site B. The obtained data were explained by the formation of local lower symmetry domains of α- (*P*4_1_22/*P*4_3_22) or β-type (*Imma*) having a size of ≤50 lattice constants. The domains can be organized in a twinned motif with the I, II, III, IV, V, or VI orientation for the α- and β-type, respectively. While the α-type is fully supported by the observed number of Raman bands and polarization rules, the β-type satisfies only a fraction of the obtained bands; however, it cannot be completely ruled out due to good agreement between calculated and observed bands at specific frequencies. Furthermore, the possible existence of both types of domains in superposition together with twinning complicates data interpretation. O’Quinn et al. (2020) proposed the Pauling rule as a base to understand the SRO phenomena in spinel, weberite, and pyrochlore structures. According to Pauling’s second rule, the sum of the bond strengths (a charge divided by coordination number) of the cations should be equal to the charge of the anion. In the normal spinel structure, each anion X is surrounded by three octahedrally coordinated cations B and one tetrahedrally coordinated cation A (Figure 8B). In the oxide spinel MgAl_2_O_4_, such arrangement leads to the sum of bond strengths 2.0, which is in excellent agreement with Pauling’s second rule. When inversion occurs, the number of possible anion coordinations extends to eight (Figure 8B). However, in MgAl_2_O_4_, only two of these configurations (with bond strength 2.083 and 1.917) are close to satisfying Pauling’s second rule and suggesting preference for these local clusters with such ion arrangement in the disordered MgAl_2_O_4_ samples (O’Quinn et al., 2020).

## Discussion: Why Do Different Structures Have Different Modes of Disorder?

It is clear that, for any significant disorder to exist, the structure has to be flexible enough to accommodate it without exorbitant energetic penalty. In general, defects and disorder raise energy and entropy, and the balance of these terms determines the equilibrium extent of disorder at a given composition, temperature, and pressure. In largely ionic oxide systems, cation size and charge are major factors determining both structure and defect chemistry. In comparing the four groups of structures discussed here, one can draw the following inferences. The fluorite-derived structures are the most tolerant of disorder on both cation and anion sublattices. Its aristotype structure consists of alternating occupied and unoccupied cubes of anions (Subramanian et al., 1983; Chakoumakos, 1984; Ewing et al., 2004; Gardner et al., 2010). This means that a change in coordination or bonding in one filled cube has relatively little influence on the next filled cube because they do not directly share anion vertices. This structural feature may explain the easy formation of oxygen vacancies and the wide homogeneity ranges in AO_2_-BO_1.5_ systems like YSZ, but it does not readily explain the wealth of different ordered phases and the overwhelming evidence for clustering at various length scales. Such clustering and/or ordering involves cooperative interactions involving the lattice dynamics (and in some cases electron delocalization) at length scales of nanometers to micrometers, not just those involving nearest and next nearest-neighbor interactions. The wealth of structural phase transitions involving changes in symmetry but having very small thermodynamic consequence (enthalpies of transformation less than 5 kJ/mol in magnitude) in both fluorite and perovskite structures speaks to the closely balanced energetics and lattice dynamics of these structures.

The thermodynamic favorability of disordering has been associated with a decrease in the cation radii ratio (r_A_/r_B_) for most of the fluorite-related systems. However, this trend is not observed in rare earth titanate and stannate pyrochlore systems, RE_2_M_2_O_7_ (M = Ti and Sn), in which only the ordered structure has been observed for all compositions all the way to RE = Yb, the smallest rare earth ion (Kennedy, 1996; Baroudi et al., 2015). One reason for such preference for the ordered structure might be the coordination geometry around Ti and Sn cations. They tend to prefer octahedral coordination as found in their natural binary oxide minerals TiO_2_ and SnO_2_ which crystallize in the rutile structure (Burdett et al., 1987; Bolzan et al., 1997). The ordered structure offers six-coordinated geometry for Ti and Sn cations, whereas seven-coordinated geometry is found for all cations in the disordered structure. The stronger covalency associated with tin may also be a factor favoring the ordered state.

For the idealized structures, configurational entropy increases in the order pyrochlore, weberite, and defect fluorite as disorder increases. The existence of short-range weberite domains within both amorphized and defect fluorite structures and their persistence into recrystallized pyrochlore is still somewhat a mystery and may represent a combination of thermodynamic and kinetic effects. The thermodynamic driver may be a compromise between energy and entropy, but the size distribution of the domains and interactions at their interfaces with the host structure (amorphous, defect fluorite, or pyrochlore) are still poorly known.

From the structural point of view, though the fully disordered structure has common seven coordination geometry for A and B cations and randomness for vacancies, the bigger A and smaller B cations prefer higher and lower coordination, respectively, leading to ordering of vacancies and consequently to locally ordered structure in nanoscale domains (Shamblin et al., 2016a). The occurrence of weberite nanodomains, which do not grow to macroscopic size and which represent a structure not seen in the equilibrium phase diagram, appears to be a unique feature of pyrochlore systems. From the kinetic point of view, annealing of such domains still needs study. Nor is it clear why certain A_2_B_2_O_7_ materials crystallize as pyrochlore, others were weberite, though in both cases, as the cations become more similar, the defect fluorite structure becomes more favorable and may occur as a high-temperature equilibrium phase. The preference of A_2_B_2_O_7_ and A_3_BO_7_ for pyrochlore and weberite structures, respectively, may reflect the different cationic charge and resulting differences in electrostatic energies, but these factors have not been explored quantitatively. In terms of coordination geometry and distortions, the weberite structure appears to be more flexible than the pyrochlore structure by accepting cations in different coordination geometries (six to eight) and being more tolerant of polyhedral distortions (Cai and Nino, 2009; Cai and Nino, 2011). This flexibility may be more important when the cation charge difference is greater.

Furthermore, the fluorite-based materials do not readily form layered or two-dimensional superstructures, and the oxygen vacancies are not coalesced into shear planes or ordered CS structures. Why are such structures unfavorable? One of the reasons may be that the vacancies tend to be separated from each other to reduce the destabilization due to electrostatic repulsion as the fluorite structure is compact without an open framework. Also, when the vacancies in fluorite-related systems are increased to stoichiometry A_2_B_2_O_6_, the bixbyite structure with ordered vacancies forms (King et al., 2013). The bixbyite structure may “outcompete” other arrangements, including shear structures, in energy. Thus, the lack of the latter may reflect the existence of an even better structural arrangement rather than any intrinsic instability of shear. A computational approach to the stability and defect chemistry of these different hypothetical polymorphs is likely to be informative.

Perovskites are also tolerant of oxygen vacancies, but these often aggregate into specific ordered and layered structures or are essentially eliminated in CS structures. Within the perovskites, the framework of corner-linked octahedra is strongly bound yet flexible. The oxygen sublattice is far from being close-packed. In terms of distortions, both tilting of octahedra and off-centering of cations within them are mechanisms to adjust bond lengths. Much of this behavior is captured by the geometric arguments embedded in the Goldschmidt tolerance factor. Phase transitions involving these distortions are generally small in energy and entropy, and the more symmetrical structures occur with increasing temperature. If the volume of the perovskite increases with increasing symmetry, as is often the case, then the pressure will extend the temperature stability field of the more disordered structures.

From the thermodynamic point of view, the annihilation of oxygen vacancies should be an energetically favorable process since vacancy formation is energetically expensive. Following this argumentation, shear plane formation, involving vacancy elimination, should be a more prevalent type of defect accommodation in different crystal structures. However, it is not common, and one should also consider that the metal-metal distances become shorter in shear planes compared with their original positions. Therefore, to form shear planes, the exothermic energy released from annihilation of oxygen vacancies should be larger than the endothermic energy of cation repulsions in shear planes. So far, only a few oxides based on ReO_3_ and rutile structures have been reported to form ordered shear structures. Based on this knowledge, it appears that the flexibility of the framework, some structural openness rather than close packing, high polarizability of atoms, octahedral coordination of metal atoms, and presence of corner-shared oxygen appear to all be essential for the formation of shear structures.

The clustering of vacancies into shear planes and their ordering into periodic structures diminishes the configurational entropy initially present on the anion sublattice. Presumably ordering is associated with a stabilizing energy (enthalpy) contribution and the formation of the shear phase reflects a balance between ΔH and TΔS. However, phases with ordered shear planes often appear to need another source of configurational entropy to stabilize them. This may arise from cation disorder in regions between the shear planes (Voskanyan and Navrotsky, 2021). This necessary and delicate balance of energetics within and between the shear planes may contribute to the relative rarity of shear structures.

The rocksalt and spinel structures contain (almost) cubic close-packed anion sublattices. Oxygen vacancies do not occur at appreciable levels but cation vacancies (and in some cases interstitials) are relatively easily accommodated. Charge balance occurs largely through variation in TM oxidation state. The cations are generally more similar in size than those occupying A- and B-sites in fluorite derivatives and perovskites. Though to a first approximation the ions on each sublattice are randomly mixed, there is increasing evidence of short-range order. Such local ordering, only beginning to be studied in detail, may suggest possible complexities in rocksalt and spinel structures analogous to those first discovered in pyrochlores and perovskites.

The discussion above reminds us how little we still know about short and midrange ordering, which appears to be a very general phenomenon in oxides. Cation radius and coordination number, as well as the openness and flexibility of the framework, appear to be the major factors influencing defect chemistry. To complement empirical trends and observations, rigorous first-principles calculations comparing different structures at the same composition would provide insight into the stability and defect chemistry in different, heretofore unsynthesized, polymorphs.

## Conclusion

Fluorite-derived phases include a number of different structures, with high oxygen vacancy concentrations and varying degrees of cation disorder. SRO leading to cluster formation and nanoscopic weberite domains are common themes. The energetics of these phases and their solid solutions are complex because these materials are often neither completely ordered nor completely disordered, with the equilibrium degree of ordering depending on temperature, pressure, and composition. Nonequilibrium disorder can be produced by different means and its annealing appears to require long-term heating at temperatures above 1,200°C. The size, nature, and energetics of clusters and nanoscopic domains deserve additional systematic study.

Perovskites are very flexible in composition, cation ordering schemes, distortions, and the incorporation of oxygen vacancies. In addition to order-disorder on both A and B cation sites, the formation of clusters involving oxygen vacancies is possible. A unique way of ordering and eliminating oxygen vacancies, not seen in fluorite structures, is the development of shear planes and their ordering into various families of CS structures, not seen in the other structure types discussed here but observed in lower symmetry structures, such as those derived from rutile. Thermodynamically, ordering into shear planes decreases the entropy associated with oxygen vacancies, but disorder in other parts of the structure may add a stabilizing configurational entropy term, perovskite structures.

Both ordered and disordered rocksalt structures form a variety of systems. It is important to realize that the relation between ordered and disordered structures definitely is not a true order-disorder transition at the same chemical composition. Rather, ordered and disordered lithium TM oxides form having an entirely different composition. It is clear that a number of factors related to cation size and the nature of the TM affect the structure formed at a given composition. In contrast to fluorite and perovskite structures, oxygen vacancies do not play a significant role in either ordered or disordered rocksalt oxides. However, distortion of polyhedra and short-range order are important in nominally DRO. Much work remains to be done to understand these changes on an atomistic level and to optimize structures and compositions for battery operation.

Order-disorder in spinels is dominated by the exchange (disordering) of cations between octahedral and tetrahedral sites. Though interstitial cations may sometimes exist, oxygen vacancies are generally not incorporated. It is interesting that spinels, long considered to be “simple” in the sense of random cation arrangements on each sublattice, may in fact harbor ordering on different length scales analogous to that seen in fluorite, perovskite, and rocksalt phases.

## Data Availability

The original contributions presented in the study are included in the article/supplementary files; further inquiries can be directed to the corresponding author.
